# Is It Time to Redefine Clinical Nodal Staging for Breast Cancer?

**DOI:** 10.1245/s10434-026-20128-y

**Published:** 2026-07-16

**Authors:** Pooja M. Varman, David A. Taft, Monik Patel, Rahul Rangan, Regina Matar-Ujvary, Caitlin Ilkanich, Zahraa Al-Hilli

**Affiliations:** 1 Department of General Surgery, Digestive Disease Institute, Cleveland Clinic, Cleveland, OH USA; 2 Department of Diagnostic Radiology, Imaging Institute, Cleveland Clinic, Cleveland, OH USA; 3 Breast Center, Integrated Surgical Institute, Cleveland Clinic, Cleveland, OH USA

**Keywords:** Clinical nodal staging, Breast cancer, Neoadjuvant chemotherapy, Pathologic nodal burden, Axillary imaging, Sentinel lymph node biopsy, Upstaging

## Abstract

**Background:**

The current American Joint Committee on Cancer (AJCC) staging system defines cN1 by node mobility rather than number, while pathologic staging stratifies by node count. Whether cN1 patients with four or more suspicious nodes on pretreatment imaging differ in pathologic stage and survival outcomes from those with fewer nodes is unclear.

**Patients and Methods:**

Patients with biopsy-proven cN1 or cN2a breast cancer diagnosed in 2018–2023 who received neoadjuvant chemotherapy (NAC) at a single institution were identified. Pretreatment imaging was reviewed and cases categorized as cN1a (1–3 mobile nodes), cN1b (≥ 4 mobile nodes), or cN2a (matted nodes). Clinicopathologic characteristics were compared using *χ*^2^ and Kruskal–Wallis tests. DFS and OS were estimated with Kaplan–Meier curves and multivariable Cox regression.

**Results:**

Among 618 cases, 390 (63.1%) were cN1a, 166 (26.9%) cN1b, and 62 (10.0%) cN2a. Pathologic complete response (pCR) rates did not differ across groups (29.5%, 28.9%, 35.5%; *P* = 0.598). Among patients with residual nodal disease, mean positive nodes were 2.8, 4.6, and 5.5 in cN1a, cN1b, and cN2a (*P* < 0.001), respectively. Pathologic nodal stage was ypN2 or higher in 12.8%, 24.1%, and 29.0% (*P* = 0.001), and cN1b was independently associated with upstaging on multivariable analysis (OR 1.958, 95% CI 1.184–3.237, *P* = 0.009). At median follow-up of 41.4 months, 3-year DFS (88.0%, 89.0%, 80.6%; *P* = 0.949) and OS (91.6%, 92.3%, 91.6%; *P* = 0.947) did not differ across groups.

**Conclusions:**

In cN1 breast cancer, imaging-defined nodal burden ≥ 4 nodes independently predicts upstaging and greater residual nodal burden after NAC, aligning pathologically with cN2a disease. Imaging-based subcategorization of cN1 disease may have implications for staging, surgical planning, and trial eligibility.

Nodal status remains one of the most important prognostic factors in breast cancer, with increasing lymph node involvement strongly correlating with worse survival outcomes.^1–3^ The current American Joint Committee on Cancer (AJCC) staging system distinguishes clinical nodal categories (cN) from pathologic nodal categories (pN) on fundamentally different criteria: pathologic staging stratifies patients by node location and the number of involved lymph nodes, while clinical staging is defined by node location and mobility, irrespective of the number of nodes involved.^4^ Clinical nodal staging has meaningful implications for how patients are risk-stratified prior to treatment, while pathologic nodal staging impacts adjuvant systemic therapy recommendations. Among patients with nodal disease, those with four or more pathologically positive nodes (pN2a) demonstrate substantially worse cause-specific survival compared with patients with one to three positive nodes (pN1a).^3,5^ The granularity of the pN staging system reflects this positive correlation between increasing lymph node involvement and worse outcomes. Modern breast imaging is routinely performed preoperatively and has demonstrated diagnostic value in identifying nodes suspicious for metastatic involvement.^6–8^ Multiple studies have shown that the number of suspicious nodes identified on axillary ultrasound and magnetic resonance imaging (MRI) correlates with pathologic nodal burden at surgery.^9–11^ Per current American College of Radiology (ACR) guidelines, however, the number of abnormal-appearing nodes is not routinely reported and does not influence cN stage.^6,12^ Whether the number of suspicious nodes detected on pretreatment imaging in patients treated with neoadjuvant chemotherapy (NAC) adds prognostic information beyond the binary cN1 versus cN2 designation is not well established. If cN1 patients with higher nodal burden on pretreatment imaging have pathologic outcomes more similar to cN2a patients, the current staging framework may underestimate risk in a meaningful subset of cN1 patients, which could have implications for surgical planning, adjuvant therapy decisions, and trial eligibility. We sought to assess differences in pathologic stage and survival outcomes in patients treated with NAC with cN1 disease with four or more suspicious lymph nodes on pretreatment imaging compared with cN1 with one to three nodes or cN2a patients.

## Patients and Methods

### Study Design and Cohort

This study was approved by the Cleveland Clinic Institutional Review Board. Patients diagnosed with biopsy-proven node-positive breast cancer at a single institution between January 2018 and December 2023 were identified. Eligible patients had clinical stage I–III breast cancer with cN1 or cN2a disease, received NAC, had pretreatment imaging available for review and underwent surgery at the same institution. Patients with bilateral breast cancer were included, with each side logged as a separate case when the contralateral breast independently met eligibility criteria. Patients were excluded if they had metastatic disease, cN2b disease (internal mammary nodal involvement), or if they received neoadjuvant endocrine therapy alone without NAC.

### Imaging Review and Nodal Categorization

Of patients who met eligibility criteria, pretreatment imaging studies were reviewed independently by two radiologists blinded to pathologic outcomes. Eligible imaging included axillary ultrasound, breast MRI, and chest computed tomography (CT) performed as part of the workup. For each patient, the total number of axillary lymph nodes with suspicious imaging features (cortical thickening defined as greater than 3 mm, loss of fatty hilum, abnormal morphology, or pathologic size) was counted across all available modalities. The final node count was determined by the modality demonstrating the greatest number of suspicious nodes. For any discordant or equivocal node counts, a third radiologist reviewed the available imaging to adjudicate.

On the basis of pretreatment imaging findings and documentation of the clinical examination at presentation, patients were categorized into three groups: cN1a (cN1 disease with 1–3 mobile suspicious nodes), cN1b (cN1 disease with 4 or more mobile suspicious nodes), and cN2a (fixed or matted axillary nodes). The cN1a and cN1b designations were created for the purpose of this study to reflect imaging-based subcategorization of patients who met the existing AJCC cN1 definition. The cN2a group served as a reference group with a higher burden of nodal disease.

### Data Collection

Patient demographics, tumor characteristics, and treatment information were obtained through an institutional tumor registry and chart review. Tumor characteristics collected included clinical T category (cT), Nottingham histologic grade, tumor histology (invasive ductal carcinoma [IDC], invasive lobular carcinoma [ILC], mixed, or other), and receptor subtype (hormone receptor-positive/HER2-negative [HR+/HER2−], HR+/HER2+, HR−/HER2+, and triple-negative breast cancer [TNBC]). Treatment variables included the type of axillary surgical procedure performed (sentinel lymph node biopsy [SLNB], SLNB with conversion to completion axillary lymph node dissection [ALND], upfront ALND) and adjuvant radiation.

Pathologic outcomes were recorded from final surgical pathology reports, including pathologic T category (ypT) and N category (ypN). Pathologic complete response (pCR) was defined as the absence of residual invasive disease in both the breast and axilla (ypT0 or ypTis, ypN0).^13^ Nodal pCR was defined as ypN0 regardless of breast response. Upstaging for cN1 cases was defined as a final ypN category of ypN2 or higher following NAC. For patients with residual nodal disease, the total number of pathologically positive nodes was recorded.

Survival outcomes were obtained through chart review. Disease-free survival (DFS) was defined as the time from the date of diagnostic biopsy to the date of first locoregional or distant recurrence, or death from any cause, whichever occurred first. Overall survival (OS) was defined as the time from the date of diagnostic biopsy to the date of death from any cause. Patients without an event were censored at the date of last follow-up.

### Statistical Analysis

Descriptive statistics were reported as counts and percentages for categorical variables and as medians with interquartile ranges (IQR) for continuous variables. Differences across the three nodal groups were assessed using the chi-squared (*χ*^2^) test for categorical variables and the Kruskal–Wallis test for continuous variables. Statistically significant omnibus results were followed by pairwise comparisons to identify group-level differences. Survival distributions were estimated using the Kaplan–Meier method, and log-rank tests were used to compare DFS and OS across nodal groups.

Multivariable Cox proportional hazards models were constructed to evaluate the independent association between nodal group and axillary management and time-to-event outcomes. Covariates included in all models were determined a priori and consisted of age at diagnosis, sex, race and ethnicity, tumor type, tumor subtype, Nottingham grade, and clinical T stage. Results are reported as hazard ratios (HR) with 95% confidence intervals (95% CI). All statistical analyses were performed using IBM SPSS Statistics (version 29.0; IBM Corp., Armonk, NY). All tests were two-tailed, and a *P*-value of less than 0.05 was considered statistically significant.

## Results

### Patient and Tumor Characteristics

A total of 618 cases of node-positive breast cancer subsequently treated with NAC between January 2018 and December 2023 were identified and met inclusion criteria. On the basis of pretreatment imaging review, 390 cases (63.0%) were categorized as cN1a (cN1 with 1–3 suspicious nodes), 166 (26.8%) as cN1b (cN1 with ≥ 4 suspicious nodes), and 62 (10.0%) as cN2a. Final node count was determined by MRI alone in 40.3% (*n* = 224) of cases, ultrasound (US) alone in 26.1% (*n* = 145), CT alone in 0.2% (*n* = 1), and combined MRI and US in 33.5% (*n* = 186).

Median age of the cohort was 56 years (IQR 45.5–66.5 years) and differed across groups: 57 years (IQR 47–67 years) in cN1a, 53 years (IQR 43–63 years) in cN1b, and 54 years (IQR 42–66 years) in cN2a (*P* = 0.050). Clinical tumor category also differed across groups (*P* = 0.024), with cT2 disease representing the most common presentation in cN1a (*n* = 198, 50.8%) and cN1b (*n* = 81, 48.8%), while cN2a patients had a higher proportion of cT3 and cT4 tumors (*n* = 19, 30.6% and *n* = 11, 17.7%, respectively). Nottingham grade, tumor type, and tumor subtype did not differ significantly across groups (Table 1). Majority of tumors were grade 3 (*n* = 238, 61.0%; *n* = 105, 63.3%; and *n* = 41, 64.5% in cN1a, cN1b, and cN2a, respectively; *P* = 0.886) and IDC histology (*n* = 351, 90.0%; *n* = 140, 84.3%; *n* = 52, 83.9%; *P* = 0.134). Tumor subtypes were distributed similarly across groups, with HR+/HER2− representing the most common subtype in all three (*n* = 162, 41.5%; *n* = 78, 47.0%; *n* = 26, 41.9%; *P* = 0.484).

**Table 1:** Patient, tumor, and treatment characteristics and outcomes for cN1a (1–3), cN1b (4+), and cN2a

Patient and tumor characteristics	cN1a (1–3) *n* = 390 (63.1%)	cN1b (4+) *n* = 166 (26.9%)	cN2a *n* = 62 (10.0%)	*P*-value
Median age at diagnosis [IQR]	57 [47–67]	53 [43–63]	54 [42–66]	0.050
Female	388 (99.5)	164 (98.8)	62 (100)	0.518
Race and ethnicity				0.830
White	282 (72.3)	118 (71.1)	43 (69.4)	
Black or African American	79 (20.3)	31 (18.7)	13 (21.0)	
Other	29 (7.4)	17 (10.2)	6 (9.7)	
Clinical T category				**0.024**
1	63 (15.9)	14 (8.4)	11 (17.7)	
2	198 (50.8)	81 (48.8)	21 (33.9)	
3	86 (22.1)	50 (30.1)	19 (30.6)	
4	43 (11.0)	21 (12.7)	11 (17.7)	
Grade				0.886
1	11 (2.8)	6 (3.6)	1 (1.6)	
2	141 (36.2)	55 (33.1)	21 (33.9)	
3	238 (61.0)	105 (63.3)	41 (64.5)	
Tumor type				0.134
IDC	351 (90.0)	140 (84.3)	52 (83.9)	
ILC	16 (4.1)	16 (9.6)	6 (9.7)	
Mixed	20 (5.1)	10 (6.0)	4 (6.5)	
Other	3 (0.8)	0 (0)	0 (0)	
Tumor subtype				0.484
HR+/HER2−	162 (41.5)	78 (47.0)	26 (41.9)	
HR+/HER2+	88 (22.6)	31 (18.7)	9 (14.5)	
HR−/HER2+	52 (13.3)	16 (9.6)	9 (14.5)	
TNBC	88 (22.6)	41 (24.7)	18 (29.0)	

Significant values (*P* < 0.05) shown in bold.

*IQR* interquartile range, *IDC* invasive ductal carcinoma, *ILC* invasive lobular carcinoma, *TNBC* triple-negative breast cancer

### Axillary Management

Axillary surgical procedure sequence varied significantly across groups (*P* < 0.001), driven primarily by the cN2a group, in which upfront ALND without prior SLNB was performed in 48.4% (*n* = 30) of patients compared with 20.5% (*n* = 80) and 24.1% (*n* = 40) in cN1a and cN1b, respectively (Table 2). In contrast, the majority of cN1a (*n* = 310, 79.5%) and cN1b (*n* = 126, 75.9%) patients underwent SLNB as the initial axillary procedure (*P* < 0.001 versus cN2a). Among patients who underwent upfront SLNB, conversion to completion ALND did not differ significantly across groups: 44.8% (*n* = 139) in cN1a, 48.4% (*n* = 61) in cN1b, and 34.4% (*n* = 11) in cN2a (*P* = 0.358). When examining the final axillary procedure performed—combining upfront ALND with SLNB that converted to completion ALND—rates of any ALND were similar across all three groups: 56.2% (*n* = 219) in cN1a, 60.8% (*n* = 101) in cN1b, and 66.1% (*n* = 41) in cN2a (*P* = 0.254). Among cN1 patients specifically, the final axillary procedure did not differ between cN1a and cN1b (56.2% versus 60.8% any ALND, *P* = 0.306). Most patients received adjuvant radiation with no significant difference across groups: 83.8% (*n* = 327) in cN1a, 86.7% (*n* = 144) in cN1b, and 87.1% (*n* = 54) in cN2a (*P* = 0.602). Rates of lymphovenous bypass were also similar across groups: 24.2% (*n* = 53) in cN1a, 28.7% (*n* = 29) in cN1b, and 24.4% (*n* = 10) in cN2a (*P* = 0.811).

**Table 2:** Axillary management and pathologic outcomes for cN1a (1–3), cN1b (4+), and cN2a

Axillary management and pathologic outcomes	cN1a (1–3) *n* = 390	cN1b (4+) *n* = 166	cN2a *n* = 62 (10.0%)	*P-*value (cN1a versus cN1b*)*	*P*-value (cN1b versus cN2a)	*P*-value overall
Axillary procedure, final				0.306	0.464	0.254
SLNB	171 (43.8)	65 (39.2)	21 (33.9)			
ALND	219 (56.2)	101 (60.8)	41 (66.1)			
Axillary procedure, sequence				0.513	**< 0.001**	**< 0.001**
SLNB	171 (43.8)	65 (39.2)	21 (33.9)			
SLNB + ALND	139 (35.6)	61 (36.7)	11 (17.7)			
ALND	80 (20.5)	40 (24.1)	30 (48.4)			
pCR	115 (29.5)	48 (28.9)	22 (35.5)	0.892	0.339	0.598
pCR in nodes	200 (51.3)	79 (47.6)	30 (48.4)	0.426	0.915	0.702
Post-neoadjuvant therapy N category (ypN)				**< 0.001**	0.526	**< 0.001**
0	200 (51.3)	79 (47.6)	30 (48.4)			
1	140 (35.9)	47 (28.3)	14 (22.6)			
2	44 (11.3)	28 (16.9)	10 (16.1)			
3	6 (1.5)	12 (7.2)	8 (12.9)			
ypN2 or higher after NAC	50 (12.8)	40 (24.1)	18 (29.0)	**< 0.001**	0.446	**< 0.001**
Average positive nodes in those with residual tumor in nodes [SD]	2.79 [2.76]	4.61 [4.37]	5.46 [4.86]	**< 0.001**	0.345	**< 0.001**

Significant values (*P* < 0.05) shown in bold.

*SLNB* sentinel lymph node biopsy, *ALND* axillary lymph node dissection, *pCR* pathological complete response, *SD* standard deviation, *NAC* neoadjuvant chemotherapy

### Pathologic Outcomes

Rates of overall pCR did not differ significantly across nodal groups (cN1a *n* = 115, 29.5%; cN1b *n* = 48, 28.9%; cN2a *n* = 22, 35.5%; *P* = 0.598). Similarly, nodal pCR (ypN0) rates were comparable across groups (*n* = 200, 51.3%; *n* = 79, 47.6%; *n* = 30, 48.4; *P* = 0.702) (Table 2). However, the distribution of post-NAC nodal category (ypN) differed significantly across groups with higher rates of ypN2 and ypN3 disease in cN1b and cN2a compared with cN1a (*P* < 0.001). Among patients with residual nodal disease, the mean number of pathologically positive nodes increased significantly with pretreatment nodal category: 2.8 in cN1a, 4.6 in cN1b, and 5.5 in cN2a (*P* < 0.001).

Upstaging to ypN2 or higher occurred in 12.8% (*n* = 50) of cN1a patients, 24.1% (*n* = 40) of cN1b patients, and 29.0% (*n* = 18) of cN2a patients (*P* = 0.001). Pairwise comparisons demonstrated that the mean number of pathologically positive nodes and rates of ypN2 or higher staging were similar between cN1b and cN2a, and both were significantly greater than cN1a (*P* < 0.001).

On multivariable logistic regression analysis among cN1 patients, cN1b disease was independently associated with upstaging to ypN2 or higher after NAC compared with cN1a (OR 1.958, 95% CI 1.184–3.237, *P* = 0.009), after adjusting for age, sex, race and ethnicity, histology, tumor subtype, grade, and clinical T stage. Tumor subtype was also a significant independent predictor of upstaging (*P* < 0.001), with HER2+ subtypes demonstrating substantially lower odds of upstaging compared with HR+/HER2− disease (HR+/HER2+: OR 0.141, 95% CI 0.054–0.370; HR−/HER2+: OR 0.201, 95% CI 0.068–0.591).

### Recurrence

At a median follow-up 41.4 months (IQR 27–60 months), recurrence occurred in 77 of 618 cases (12.5%). Recurrence rates were similar across nodal groups: 49 patients in cN1a (12.6%), 19 in cN1b (11.4%), and 9 in cN2a (14.3%). The majority of recurrences were distant across all groups: 43 (11.0%), 16 (9.6%), and 5 (8.1%) in cN1a, cN1b, and cN2a, respectively. Locoregional recurrence was uncommon in cN1a (*n* = 6, 1.5%) and cN1b (*n* = 3, 1.8%) but occurred at a higher rate in cN2a (*n* = 4, 6.5%), though this difference did not reach statistical significance given the small number of events.

### Survival Outcomes

**Fig. 1:**
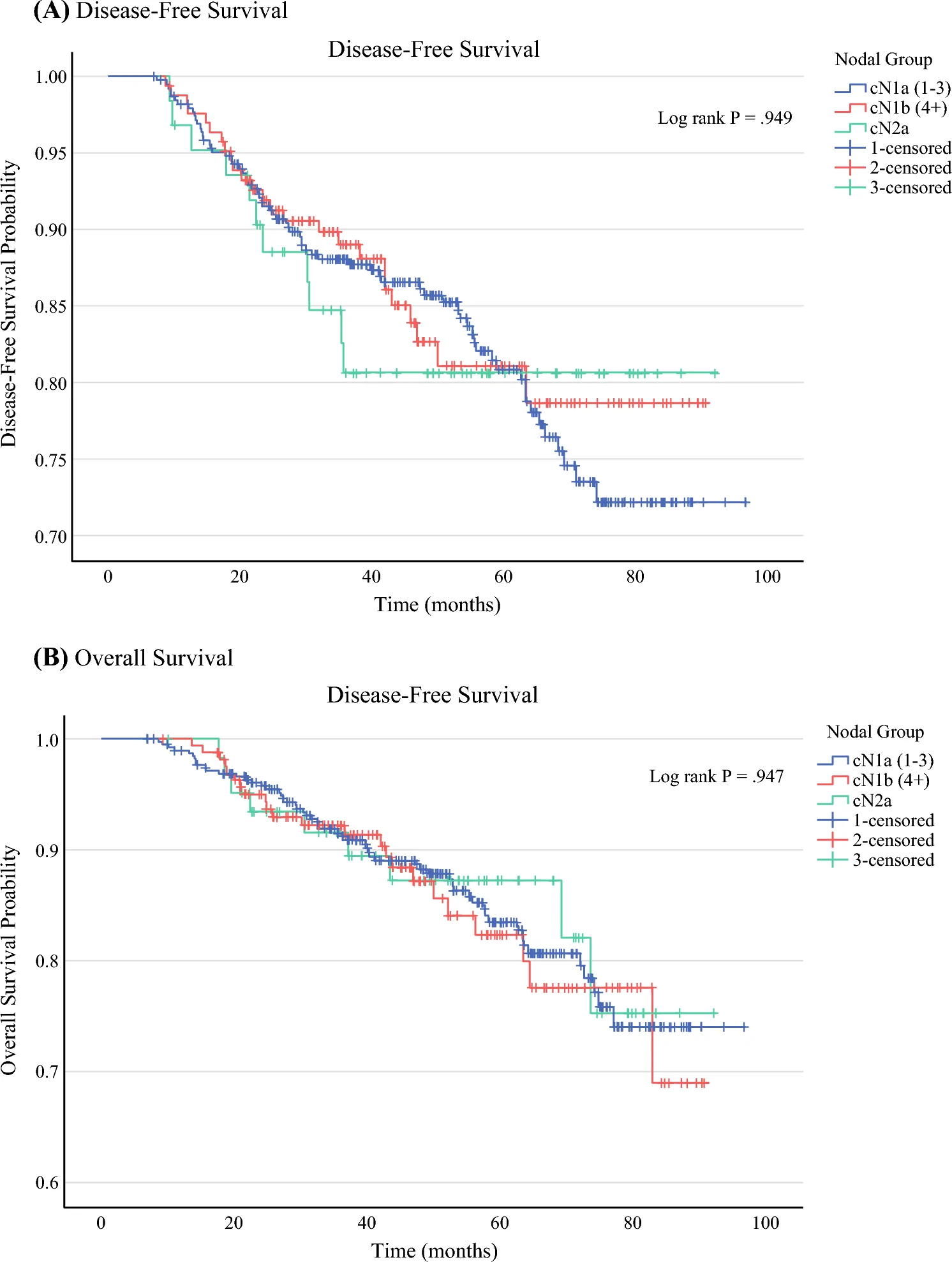
Kaplan–Meier curves for (**A**) disease-free survival and (**B**) overall survival

Estimated 3-year DFS was 88.0% (95% CI 84.7–91.3) in cN1a, 89.0% (95% CI 84.1–93.9) in cN1b, and 80.6% (95% CI 70.2–91.0) in cN2a (log-rank *P* = 0.949). Estimated 3-year OS was 91.6% (95% CI 88.7–94.5), 92.3% (95% CI 88.2–96.4), and 91.6% (95% CI 84.5–98.7), respectively (log-rank *P* = 0.947). Kaplan–Meier curves for DFS and OS are shown in Fig. 1.

On multivariable Cox proportional hazards analysis adjusting for age, sex, race and ethnicity, histology, tumor subtype, grade, and clinical T stage, nodal group was not independently associated with DFS or OS (Table 3). Increasing age at diagnosis, tumor subtype, and clinical T stage were significant independent predictors of both outcomes. Compared with HR+/HER2− disease, HER2+ subtypes demonstrated substantially lower hazards of recurrence and death (HR approximately 0.37–0.46). Compared with T1 disease, T4 was associated with a fourfold higher hazard of death (HR 4.310, 95% CI 1.708–10.873, *P* = 0.002). Sex, race and ethnicity, histology, and grade were not significant independent predictors.

**Table 3:** Multivariable Cox proportional hazards analysis for disease-free survival (DFS) and overall survival (OS)

	**DFS**		**OS**	
Characteristic	HR (95% CI)	*P*-value	HR (95% CI)	*P*-value
Nodal group		0.778		0.755
cN1a				
cN1b	0.88 (0.55–1.40)	0.586	1.05 (0.64–1.72)	0.848
cN2a	0.84 (0.44–1.59)	0.585	0.78 (0.39–1.59)	0.501
Age at diagnosis (per year)		0.014		**< 0.001**
	1.02 (1.00–1.04)		1.03 (1.01–1.05)	
Sex		0.482		0.976
Female				
Male	2.05 (0.28–15.25)	0.482	–	0.976
Race and ethnicity		0.569		0.672
White				
Black or African American	0.87 (0.54–1.39)	0.550	0.83 (0.51–1.37)	0.474
Other	0.62 (0.23–1.68)	0.350	0.76 (0.26–2.18)	0.610
Clinical T stage		**< 0.001**		**0.013**
1				
2	2.62 (1.11–6.16)	0.028	2.32 (0.97–5.52)	0.058
3	3.61 (1.50–8.69)	0.004	2.85 (1.17–6.99)	0.022
4	5.61 (2.26–13.94)	< 0.001	4.31 (1.71–10.87)	0.002
Grade		0.699		0.659
1				
2	1.01 (0.30–3.44)	0.990	0.83 (0.24–2.91)	0.773
3	1.22 (0.35–4.22)	0.751	1.05 (0.30–3.70)	0.946
Tumor type		0.792		0.816
IDC				
ILC	0.62 (0.24–1.63)	0.333	0.59 (0.20–1.74)	0.339
Mixed	1.08 (0.48–2.43)	0.855	0.88 (0.34–2.27)	0.788
Other	–	0.959	–	0.974
Tumor subtype		**< 0.001**		**< 0.001**
HR+/HER2−				
HR+/HER2+	0.46 (0.24–0.86)	0.016	0.44 (0.21–0.91)	0.026
HR−/HER2+	0.37 (0.17–0.82)	0.015	0.40 (0.17–0.95)	0.039
TNBC	1.41 (0.90–2.22)	0.135	1.60 (0.99–2.58)	0.055

Bold *P*-values on section header rows represent overall group significance (*P* < 0.05).

– estimate not reported due to model instability (complete separation), *DFS* disease-free survival, *OS* overall survival, *HR* hazard ratio, *CI* confidence interval, *IDC* invasive ductal carcinoma, *ILC* invasive lobular carcinoma, *TNBC* triple-negative breast cancer

## Discussion

In this single-institution cohort of patients with node-positive breast cancer treated with NAC, we found that quantifying the number of abnormal lymph nodes on pretreatment imaging predicted pathologic nodal stage after chemotherapy. Patients with four or more suspicious nodes on pretreatment imaging (cN1b) were nearly twice as likely to be upstaged to ypN2 or higher after NAC compared with cN1 patients with one to three nodes (cN1a), and their mean residual nodal burden was more closely aligned with cN2a patients than with cN1a patients. The correlation between imaging node count and pathologic nodal burden has concrete implications for how cN1 disease is characterized, communicated, and incorporated into treatment planning.

The current AJCC cN framework assigns identical staging to patients with meaningfully different pretreatment disease burdens. This creates a practical problem when cN category is used as an eligibility criterion or stratification variable in clinical trials informing axillary management and radiation decisions. Several pivotal trials have used cN1 as a uniform enrollment category, including the ongoing Alliance A011202 trial evaluating axillary radiation versus completion ALND in cT1–3N1 patients with positive sentinel nodes after NAC,^14^ the RAPCHEM registry demonstrating safe radiotherapy deescalation in selected cT1–2N1 patients achieving nodal clearance after NAC,^15^ and the NRG Oncology/NSABP B-51/RTOG 1304 trial showing that omitting regional nodal irradiation in patients converting from cN1 to ypN0 did not adversely affect recurrence-free interval.^16^ Although all cN1 patients within these trials are enrolled despite the number of abnormal axillary lymph nodes on imaging, our study shows that cN1 nodal group are not a homogeneous group. Since pathologic nodal burden after NAC in our cN1b group more closely resembles cN2a disease, it may be important to risk stratify patients according to the number of abnormal lymph nodes on imaging, as done in the RAPCHEM study.

The finding that pCR rates were equivalent across all three groups (approximately 29–36%) is notable and has two important implications. First, it confirms that response to NAC is not predicted by pretreatment nodal count, but rather tumor biology. This should reassure clinicians treating higher-burden disease if the tumor subtype is likely to have good NAC response. Second, it suggests that the observed pathologic differences in residual nodal disease are driven by the subset of patients who do not achieve pCR and influenced by the extent of pretreatment disease. In that subset, the outcomes are significant: cN1b patients had on average 4.6 positive nodes at surgery compared with 2.8 in cN1a patients, a difference that carries direct implications for post-NAC axillary management. Although the rate of axillary dissection did not differ between all nodal groups, whether this practice pattern leads to undertreatment in a subset of cN1b patients warrants further investigation.

Accurate preoperative assessment of axillary nodal burden is central to these questions, and current imaging modalities offer meaningful but imperfect tools. Axillary ultrasound is widely available and allows for real-time assessment of nodal morphology, but is operator dependent and limited in its ability to evaluate level II and III nodes.^9,17^ MRI offers broader anatomic coverage and has been shown to correlate with pathologic nodal burden, though it is more resource intensive.^10,11^ The Node Reporting and Data System (Node-RADS), a recently proposed standardized framework for lymph node assessment across imaging modalities, emphasizes the importance of documenting overall disease burden and the number of abnormal nodes, in part because of the growing trend toward surgical deescalation, which demands more precise preoperative characterization of nodal involvement.^12,18–20^ Despite this, the number of suspicious nodes is not routinely reported per current ACR guidelines and does not influence cN staging.^6,12^ Our findings support the argument that routine documentation of imaging node count would provide clinically meaningful information with direct implications on application of their treatment course and pathologic disease volume.

Despite differences in residual pathologic nodal volume, nodal group was not independently associated with DFS or OS. While older women, higher clinical T stage, and triple-negative subtype were associated with worse DFS and OS ^21–28^, the absence of a nodal group survival signal should be interpreted with caution, as a median follow-up of 41.4 months is likely insufficient to detect survival differences in a predominantly HR+ cohort where late recurrences are common. Longer follow-up is necessary.

This study has several limitations. Not all imaging modalities were available for all patients, which may have introduced variability in node count determination. The cohort size limits subgroup analyses by receptor subtype or grade. Finally, as a single-institution retrospective study, findings should be validated in larger, prospective datasets.

This study contributes to a growing body of evidence that pretreatment imaging provides clinically meaningful prognostic information beyond current AJCC cN staging. The correlation between imaging node count and pathologic nodal burden is consistent and significant. Incorporating imaging-based subclassification of cN1 disease into staging systems, trial eligibility criteria, and radiologic reporting standards warrants prospective investigation.

## Conclusions

Although cN category was not an independent predictor of survival, we found that higher imaging-defined node count (≥ 4) in cN1 disease predicts greater pathologic nodal burden after neoadjuvant chemotherapy, different than those with lower cN1 node count (1–3). This study supports incorporating imaging-based subclassification of cN1 disease into the AJCC staging system and ACR radiologic reporting guidelines to further refine prognostication and trial methodologies to improve the treatment of patients with node-positive breast cancer.
